# Effects of Transcranial Direct Current Stimulation (tDCS) on Go/NoGo Performance Using Food and Non-Food Stimuli in Patients with Prader–Willi Syndrome

**DOI:** 10.3390/brainsci11020250

**Published:** 2021-02-17

**Authors:** Albert B. Poje, Ann Manzardo, Kathleen M. Gustafson, Ke Liao, Laura E. Martin, Merlin G. Butler

**Affiliations:** 1 Department of Psychiatry and Behavioral Sciences, University of Kansas Medical Center, Kansas City, KS 66160, USA; amanzardo@kumc.edu (A.M.); mbutler4@kumc.edu (M.G.B.); 2 Hoglund Biomedical Imaging Center, University of Kansas Medical Center; Kansas City, KS 66160, USA; kgustafson@kumc.edu (K.M.G.); kliao@kumc.edu (K.L.); lmartin2@kumc.edu (L.E.M.); 3 Department of Neurology, University of Kansas Medical Center, Kansas City, KS 66160, USA; 4 Department of Population Health, University of Kansas Medical Center, Kansas City, KS 66160, USA

**Keywords:** event-related potentials (ERP), transcranial direct current stimulation (tDCS), food and non-food images, Prader–Willi syndrome (PWS) and genetic subtypes, inhibition

## Abstract

Prader–Willi syndrome (PWS) is a neurodevelopmental genetic disorder characterized by multiple system involvement with hypotonia, poor suck with feeding difficulties, growth and other hormone deficiencies, intellectual disability, and behavioral problems with childhood onset of hyperphagia resulting in obesity, if not externally controlled. Transcranial direct current stimulation (tDCS) has been increasingly shown to modulate cognitive and behavioral processes in children and adults, including food-intake behaviors in patients with PWS. This study further reports the positive effects of brief tDCS sessions on Go/NoGo task performance involving food and non-food stimuli images, alterations in N2 brain amplitude, and genetic subgroup differences (maternal disomy 15, UPD; 15q11-q13 deletion, DEL) before and after tDCS as assessed by event-related potentials (ERPs) in 10 adults with PWS. The results indicate a group effect on baseline NoGo N2 amplitude in PWS patients with DEL vs UPD (*p* =0.046) and a decrease in NoGo N2 amplitude following tDCS (*p* = 0.031). Our tDCS approach also demonstrated a trend towards decreased response time. Collectively, these results replicate and expand prior work highlighting neurophysiological differences in patients with PWS according to genetic subtype and demonstrate the feasibility in examining neuromodulatory effects of tDCS on information processing in this patient population to stimulate additional research and treatment.

## 1. Introduction

Prader–Willi syndrome (PWS) is a rare neurodevelopmental genetic disorder affecting multiple systems, leading to life-threatening obesity in childhood if not externally controlled. The syndrome presents with hypotonia, feeding difficulties, developmental delay in infancy, and growth and other hormone deficiencies. The syndrome is further characterized by short stature; small hands and feet; hypogonadism/hypogenitalism; mild cognitive problems; and behavioral disturbances, including intrusive thinking patterns, outbursts, anxiety, self-injury, and risk for autism and psychosis in late adolescence and early adulthood [1,2,3,4]. The incidence of PWS affects about one in 10,000–20,000 live births, across all races and affects both genders equally [1], with 350,000 affected worldwide [5]. Lastly, PWS patients develop a pattern of food-seeking leading to hyperphagia and subsequent obesity without strict caloric restriction and food security programs in place. These behaviors also include eating of nonfood, inedible items [1,4,6].

PWS is caused by errors of genomic imprinting generally from a de novo paternal chromosome 15q11-q13 deletion (about 60% of cases), which contains imprinted genes causing the disorder. Maternal disomy 15 occurs when both chromosome 15s are inherited from the mother, which is seen in 35% of cases. The remaining individuals have a defect (microdeletion or epimutation) involving the imprinting center in chromosome 15 that controls the expression of the imprinted genes in the region or other chromosome 15 anomalies [3]. Patients with maternal disomy 15 also experience increased risk of psychiatric complications (e.g., cyclic psychosis) [1].

The interplay underlying observed neurodevelopmental contributions to hyperphagic behavior remain unclear in PWS. Evidence suggests that food-intake decisions are influenced by neural networks involving the dorsolateral prefrontal cortex (DLPFC). This structure has been implicated in the regulation and processing of food motivation, and the integration of sensory and affective information, including satiety in healthy weight controls [7,8], and has been shown to be hypoactive in PWS along with the orbitofrontal cortex when processing food stimuli [9]. This may reflect a dysfunctional satiety network in PWS since a number of structures that impact DLPFC activity such as the hypothalamus, ventromedial prefrontal cortex, insula, and amygdala have shown abnormal signaling [9,10] or morphology [11].

A growing body of literature demonstrates that brain stimulation techniques such as transcranial direct current stimulation (tDCS) provide a safe, painless, inexpensive, non-restrictive, and non-invasive method to modify neuronal and cognitive functioning in brain regions of interest [12]. This technique modulates cortical activity through delivery of a weak electrical current applied to the scalp and brain parenchyma via the anode (positive) to the cathode (negative) electrodes. Therefore, the placement of electrodes can be used to target brain regions and networks. Effects of this procedure can produce increased as well as decreased cortical excitability [13,14], impact peripheral systems [15], and be used to target specific neuroanatomy responsible for cognitive processes of interest [16]. The application of this technique to the DLPFC (left and right hemispheres) has shown reduced craving for a variety of substances, including tobacco [17,18] and food [19,20], even following a single session [17,18,19]. Furthermore, targeting this region (i.e., DLPFC) has demonstrated reduction in hyperphagia in patients with PWS [21,22] assessed via psychometric assessment in previous pilot work using small samples.

The present study sought to expand neuroanatomy and function research [23,24] to predict change in response to food and non-food stimuli and investigate event-related potentials (ERPs) relevant to response-inhibition in patients with PWS using a Go/NoGo task with food vs. non-food cues assessed via ERPs. Based on prior work, tDCS was hypothesized to modulate expected patterns of ERPs to demonstrate enhanced response-inhibition associated with processing of food-related stimuli. In addition, the electroencephalogram (EEG) would also allow for the assessment of general neurophysiology associated with PWS and genetic subtypes.

## 2. Subjects and Methods

### 2.1. Participants

Ten PWS participants (6 M, 4 F) took part in this study; 4 had the 15q11-q13 deletion (DEL), and 6 had maternal disomy 15 (UPD). Six (4 M 2 F) were in the active group (2 DEL, 4 UPD), and 4 (2 M, 2 F) were in the sham group (2 DEL, 2 UPD). The average age of participants was 29.6 ± 7 yrs (30.7 ± 9 yrs, males; 28.8 ± 5 yrs, females), with a range of 19 to 44 yrs. One female participant with UPD was randomized to the active intervention arm but stopped early in the session due to intolerance of the stimulation.

### 2.2. Go/NoGo Task

**Figure 1:**
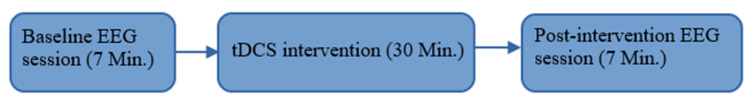
Flow diagram of study design and interventions.

The food Go/NoGo EEG task contained 160 trials with equal food (NoGo) and non-food (Go) trials. Each trial started with a food or nonfood picture displayed on the computer screen for a duration of 1 s. The picture was followed by a black screen of 1500 ms duration (varied from 1400 ms to 1600 ms) with a fixation cross in the center. The duration of the Go/NoGo task was 7 min (14 min total for pre- and post assessment). Subjects were instructed to click a mouse button in response to Go condition trials (non-food) and not respond to NoGo trials (food). All the food pictures were chosen from images used in previous studies examining brain responses to food motivation [9], and the non-food pictures were chosen from The International Affective Picture System (IAPS) [25]. The participants’ response time (unit: ms) to correct Go trials, the correct Go (hit) trials, and the mistakes in response to NoGo trials number (false alarm) were used as behavioral performance measures. After the baseline EEG session, subjects were randomized to 30-min tDCS or sham stimulation sessions. After tDCS or sham stimulation, the subjects were instructed and participated in an after-treatment EEG session to evaluate the tDCS session effect for all subjects. A flowchart of the 44-min assessment and intervention protocol is provided below (Figure 1).

### 2.3. tDCS Intervention

**Figure 2:**
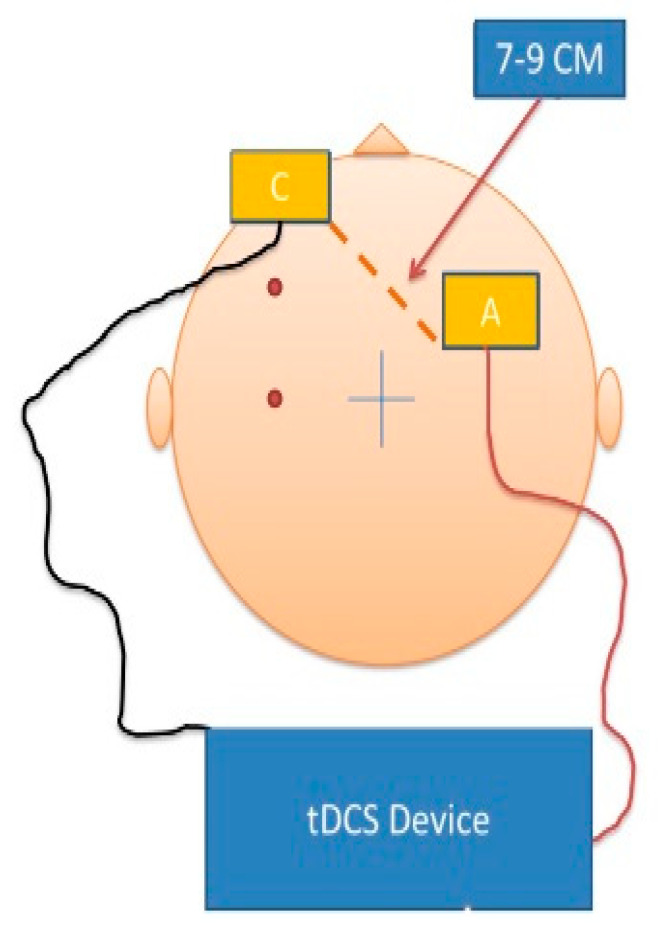
Schematic demonstrating placement of the transcranial direct current stimulation (tDCS) cathodal (C) and anodal (A) electrodes.

Participants were randomly assigned into an active or a sham tDCS session. The active tDCS intervention was applied by a low-intensity DC stimulator (Chattanooga IontoTM iontophoresis system, Chattanooga Medical Supply Inc., Chattanooga, TN, USA) that delivered 2.0 mA of direct current for 30 min through saline-soaked electrodes (35 cm^2^). The electrodes were placed in F4 for anode and Fp1 for cathode electrode, respectively, and fixed 7~9 cm apart by using rubber bands (Figure 2). Sham stimulation was delivered via brief ramp-up and ramp-down of tDC stimulation for 60 s at the beginning and end of the intervention procedure to facilitate blinding. Thus, participants in the sham condition did receive brief tDC stimulation associated with somatosensation of electrical stimulation as a reliable method for blinding reported in other studies targeting prefrontal targets [26]. Participants in both groups were asked to relax during this period and could talk with their guardians or the experimenters. The procedure was well-tolerated. All participants provided informed consent or assent in accordance with the stipulations of the Institutional Review Board (IRB) at the University of Kansas Medical Center and the Declaration of Helsinki (KUMC HSC # STUDY00141818).

### 2.4. EEG Recording and Analysis

**Figure 3:**
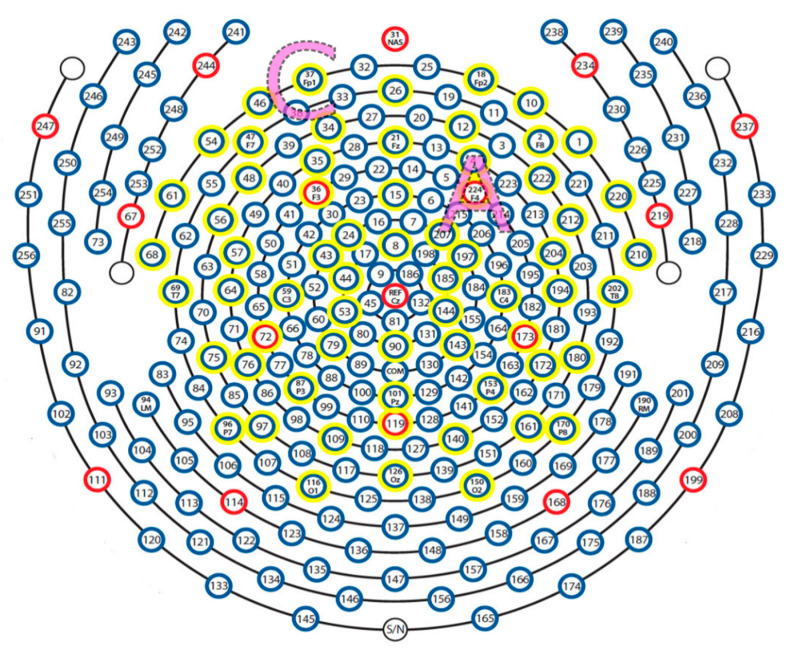
256-channel electroencephalogram (EEG) net with illustrated anatomical placement of tDCS anode (A) and cathode (C). The 64 channels used in the event-related potentials (ERP) analysis are highlighted in yellow. Anterior (top), posterior (bottom), vertex (central), left, and right, as shown in the illustration. The net was removed for the 30-min tDCS or sham intervention and replaced for the post-intervention ERP recording. The tDCS anode and cathode were placed at the site of electrodes F4 and Fp1, respectively, according to the International 10–20 EEG system.

EEG signals were recorded using a 256-channel high-density sensor net (Electrical Geodesics; EGI, Eugene, OR, USA). The vertex (Cz) electrode was used as a physical reference. EEG recordings were sampled at 1000 Hz, and data were filtered in the frequency band from 0.5 to 30 Hz and further processed in EEGLAB [27] and ERPLAB toolboxes [28]. The 256 channels were reduced to 64 channels according to the 10–20 system to decrease processing complexity. The channel layout used in recording is shown in Figure 3 with the reduced 64 channels highlighted. The Clean_rawdata plug-in with EEGLAB, which applies automated subspace removal (ASR), was used to detect and reject or remove high-amplitude non-brain activity [29]. The Adaptive Mixture Independent Component Analysis (AMICA) [30,31] was used to separate independent components. Eye, cardiac, and muscle artifacts were identified and removed using ICLabel, SASICA [28] and FASTER [32] toolboxes. Data from electrodes not passing the noise threshold were interpolated by using data from surrounding electrodes. Continuous EEG data were segmented into each trial with a length of 100-ms baseline period pre-stimulus and 1500 ms after stimulus onset.

**Figure 4:**
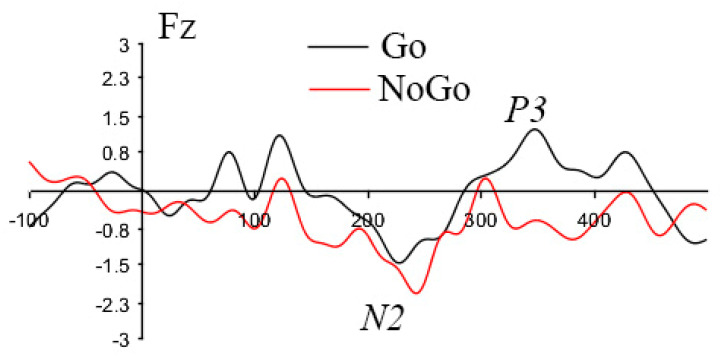
ERP waveform on Fz channels. Data are from the grand average of all subjects in the pre-treatment session.

Two inhibition-related ERP components were investigated in this study: N2 and P3. The amplitude of the N2 ERP component was defined as the minimum of individual average ERP data within the time window of 140–270 ms after stimulus onset. P3 component amplitude was defined in the window of 250–450ms in the same electrodes cluster. Fourteen frontal electrodes were chosen for both N2 and P3 analysis, which included Fp1, Fp2, Fz, F3, and F4 electrodes in the 10–20 system. The amplitude peak was computed based on the average of this electrode cluster. Only ERP data associated with a correct behavioral response were used in the statistical test analysis. The ERP waveform with N2 and P3 components on channel Fz was illustrated in Figure 4.

Significance tests were computed in order to evaluate the Go/NoGo conditions effect, tDCS treatment effect, and genetic subtype effect of both behavioral performance and ERP data. The two-tailed t-test was used and significant level set at *p* < 0.05 for all tests. The effect size was calculated using Cohen’s d to indicate the differences between means interpreted as small (d = 0.2), medium (d = 0.5), or large (d = 0.8) [33]. Due to the exploratory character of this study, the statistical significance was not corrected for multiple comparisons.

## 3. Results

### 3.1. Task Performance

Before the intervention, task performance between subjects randomly assigned to either tDCS or sham groups was tested. Subjects in the active group had evidence for significantly faster Go reaction time than the sham group (*p* = 0.054, d = 1.789); see Table 1. Task performance was also tested for differences due to genetic subtype. There was no significant difference between DEL and UPD subtype group for Go reaction time (*p* = 0.844, d = 0.108), hit rate (number of correct responses to Go stimuli) (*p* = 0.381, d = 0.503), or false alarm rate (incorrect response to NoGo) (*p* = 0.497, d = 0.588); see Table 2.

Group data for Go/NoGo task performance between and within intervention group assignment (tDCS vs. Sham) and test session (Before vs. After) are shown in Table 1. After tDCS or sham intervention, there was no between group difference in Go reaction time (*p* = 0.157, d = 1.448). Both active and sham group subjects had faster Go reaction time following their assigned intervention, but this difference was not significant in the sham group (*p* = 0.378, d = 0.515) and was only marginally significant in the active group (*p* = 0.063, d = 0.971). There was also no within-group difference between the test sessions for hit trials (active group *p* = 0.927, d = 0.031; sham group *p* = 0.226, d = 0.876) or false alarm rate (active group *p* = 1.000, d = 0.000; sham group *p* = 0.207, d = 0.927). There was no difference between groups, within the first and second test sessions, for hit trials (first *p* = 0.516, d = 0.429; second *p* = 0.549, d = 0.823), or false alarm rate (first *p* = 0.154, d= 7.139; second *p* = 0.909, d = 0.105).

### 3.2. ERP Findings

#### 3.2.1. Go vs. NoGo Condition Effects

The non-food (Go) vs. food (NoGo) images did not generate an N2 amplitude difference in either group during the first test session, in the active group (*p* = 0.311, d = 0.387) or sham group (*p* = 0.151), or in the second post-treatment test session (active *p* = 0.747, sham *p* = 0.776).

#### 3.2.2. PWS Genetic Subtype Differences

**Figure 5:**
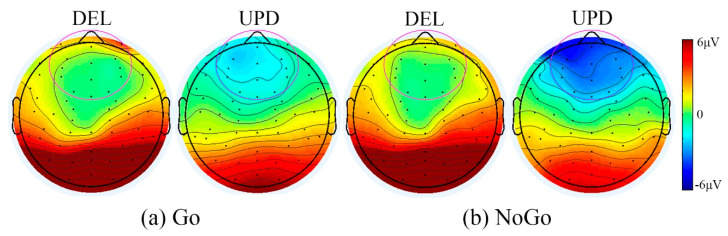
Heat maps between DEL and UPD groups on baseline Go/NoGo task. Here, a symmetrical color bar is used. The red indicates activity with positive amplitude, the blue indicates negative amplitude, and the green indicates activity with zero value. Range = +/− 6 microvolts. The channels used in ERP analysis are highlighted (t = 245ms). (**a**) Go condition when viewing non-food images. (**b**) NoGo condition when viewing food images.

The PWS genetic subtype difference was not significant for N2 amplitude in the Go condition (*p* = 0.091, DEL = −1.21uV, UPD = −3.93uV). However, as demonstrated by a group X trial type interaction, the DEL group had a significantly smaller N2 amplitude (*p* = 0.046) than the NoGo condition than the UPD group (DEL = −1.27, UPD = −4.55) during baseline Go/NoGo task. The heatmaps in Figure 5 illustrate this pattern of results.

#### 3.2.3. Treatment Effects Using tDCS

**Figure 6:**
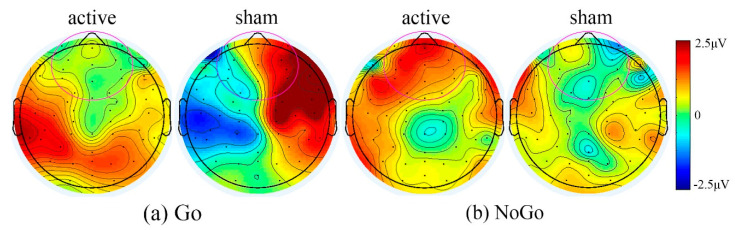
Heat map differences demonstrating decreased N2 activity on NoGo trials across groups following tDCS. Differences are calculated as tDCS treatment minus baseline. Here, a symmetrical color bar is used. The red indicates activity with positive amplitude and the blue indicates negative amplitude and the green indicates activity with zero value. Range = +/− 2.5 microvolts. The channels used in ERP analysis are highlighted (t = 173ms). (**a**) Go condition. (**b**) NoGo condition.

Treatment with tDCS or sham did not result in a significant change in N2 amplitude for the Go condition, active group (*p* = 0.634), or sham group (*p* = 0.665). However, for the NoGo condition, the active group had a significant decrease in N2 amplitude after tDCS (pre-treatment =−3.40uV, tDCS =−2.38uV) (*p* = 0.031). This difference was not seen in the sham group (pre-treatment =−3.00uV, sham =−3.22uV) (*p* = 0.834). This effect is demonstrated via heat maps in Figure 6.

## 4. Discussion

The present study sought to extend previous research findings investigating the role of DLPFC in hyperphagia behavior in PWS patients using tDCS [21,22]. As prior work investigated the role of ERPs during Go/NoGo task performance [34], it was hypothesized that tDCS of the right DLPFC may affect decision-making and response inhibition during a Go/NoGo task in PWS while processing food and non-food stimuli [21]. The tDCS montage used in the present study utilized anode electrode placement at F4 and cathode electrode placement at Fp1 to target this region using a pre-post design immediately following 30 min of tDCS intervention. Application of this technique to the DLPFC has impacted craving for a variety of substances including tobacco [17,18] and food [17,19,20]. Furthermore, targeting this region (i.e., DLPFC) has demonstrated reduction in hyperphagia in patients with PWS [21,22], making it of particular interest in this area of research.

Behavioral data demonstrated a trending effect of tDCS on response time during the Go/NoGo task. Active as opposed to sham tDCS stimulation produced maintenance of reaction time following 30 min of 2.0 mA DC to the right DLPFC. This was limited to processing-speed and did not impact accuracy of behavioral performance across genetic subtypes. This may represent evidence of increased frontal cortical excitability [13] since selective attention may have been enhanced immediately following tDCS.

Electrophysiological data demonstrated differences in stimulus processing across the DEL and UPD groups. Although N2 amplitudes were noted to be equivalent across food and non-food stimuli during the Go/NoGo task, DEL PWS patients demonstrated smaller N2 amplitude during the NoGo trials relative to the UPD group. This pattern of results may suggest decreased conflict monitoring in this group when processing food stimuli during NoGo trials [34]. Differences in neurophysiology during food-image processing across PWS subtypes have also been shown in other studies [35]. Furthermore, alterations in N2 have been seen across PWS subtypes relative to controls during continuous performance task assessments implicating differences in inhibitory function in PWS patients relative to controls [36]. These findings add to the literature highlighting baseline differences in information-processing across these PWS patient groups. Further examination into the relationship between neurophysiology, cognition, and behavior is needed to evaluate the effects of neuromodulation as a potential intervention in these patients as the PWS community continues to search for intervention protocols for hyperphagia in PWS.

Neuromodulation produced by tDCS appeared to further impact these baseline differences. Our tDCS procedure produced a significant reduction in N2 amplitude across groups associated with equivalent N2 amplitude rendered during the processing of food stimuli, thereby reducing baseline differences observed in the DEL and UPD PWS subgroups. The decreased N2 amplitude in the active group after tDCS intervention suggests that tDCS affects response inhibition in this context. This result is partially consistent with the reports by Lapenta et al. [37], who demonstrated lower N2 amplitude following tDCS in the active group when compared with sham group. In that study, active tDCS reduced N2 amplitude to food stimuli, which was associated with decreased caloric intake in control subjects. Decreased N2 activation was seen only on NoGo (food image trials) and therefore was not a generalized effect to non-food stimuli. Therefore, this pattern of results may represent potential neural activity that is associated with subsequent behavior change, which may be of significance in patients with PWS. Given the nature of hyperphagia in this patient population, this may reflect changes across studies in information-processing relating to food stimuli in the human brain that may be associated with additional non-pharmacological intervention opportunities for individuals with PWS.

Prader–Willi syndrome (PWS) is a rare genetic disorder associated with multiple physical, cognitive, and behavioral abnormalities with well-documented clinical abnormalities. Additionally, genetic and brain imaging studies have characterized neurological differences in patients with PWS as they relate to behavioral manifestations. For example, individuals with only a partial deletion of chromosome 15 at the 15q11.2 BP1-BP2 region, including four genes (*NIPA1, NIPA2, CYFIP1*, and *TUBGCP5*) have reported neurodevelopmental-autism phenotypes [3]. Previous studies have shown that about 40% of participants with PWS and the paternal 15q11-q13 deletion will have this proximal 15q11.2 BP1-BP2 region deleted known as the larger typical 15q11-q13 Type I deletion [38]. Those with this small 15q11.2 BP1-BP2 deletion only or having Burnside–Butler syndrome are reported with lower surface area of the brain, a thicker cortex and a smaller nucleus accumbens. Furthermore, regional cortical analyses show localization of the effects to the frontal, cingulate, and parietal lobes. EEG studies have documented differences in patients with maternal disomy 15 relative to those with the deletion. Maternal disomy 15 patients have significantly increased reaction times compared to deletion or healthy controls. Deficits in specific ERPs related to early modality or specific inhibition patterns have been reported during late general inhibition of N200 and P300 peaks, relative to controls [36]. These findings may have behavioral significance in relation to the PWS phenotype since patients with the small 15q11.2 BP1-BP2 deletion have lower cognitive ability in five out of seven tasks studied compared to normal controls [39], implicating genetic and neurodevelopmenal contributions to the behavioral presentation of patients with PWS [3]. Future study of intervention strategies, such as tDCS are needed.

## 5. Conclusions

Patients with PWS have a rare neurodevelopmental genetic disorder affecting multiple systems which can lead to life-threatening obesity in childhood, if not treated [1,2,3,4]. tDCS is emerging as a non-invasive method to modify neuronal and cognitive functioning associated with targeted behavioral change [17,18,19,20]. Although this pilot study employed a small sample size that requires replication, the primary findings support positive effects of brief tDCS sessions on Go/NoGo task performance involving food and non-food stimuli images as evidenced by alterations in N2 in patients with PWS following intervention. It represents a proof of concept that targeted modulation of dysfunctional brain regions associated with attention and response inhibition may be associated with hyperphagia behavior in PWS. This investigation extends prior work we reported targeting this area of DLPFC with tDCS using behavioral and psychometric measures to examine stimulation effects on hyperphagia [21,22]. Because this study examined modulators of response-inhibition primarily, future studies may benefit from additional markers of cognitive and behavioral changes associated with food motivation and intake in this patient population. Examination of gender differences is also encouraged since prior work has demonstrated that males may have differential responses to tDCS when cognitive measures are assessed [40]. Lastly, this study demonstrated baseline neurophysiological differences in how patients with PWS process food and non-food stimuli as a function of PWS genetic subtype consistent with prior studies [35,36]. Additional studies should work to examine larger groups of genetic subtype patients to better understand cognitive and neurophysiological processes associated with appraisal, motivational, and behavioral regulation as they relate to hyperphagia in PWS. This research may lead to a better understanding of hyperphagia in PWS, brain functioning, and possibly improved pathways for treatment modalities.

## Figures and Tables

**Table 1:** Task performance of intervention groups and test sessions.

Before tDCS or Sham Intervention				After tDCS or Sham Intervention			
Group	Reaction Time (ms)	Hit Rate (*n*)	False Alarm (*n*)	Group	Reaction Time (ms)	Hit Rate (*n*)	False Alarm (*n*)
Active	633.7 (106.6) *	75.3 (3.3)	3.8 (4.1)	Active	563.8 (67.0)	75.0 (8.8)	3.8 (6.0)
Sham	865.2 (160.4) *	70.3 (11.84)	0.8 (0.4)	Sham	802.3 (254.7)	77.8 (3.3)	4.3 (4.0)

* Between group, within test session; *p* = 0.054.

**Table 2:** Task performance between genetic subtype—before tDCS intervention.

Group	Reaction Time (ms)	Hit Rate (*n*)	False Alarm (*n*)
DEL	714.6 (39.1)	76.0 (2.94)	4.0 (6.1)
UPD	734.1 (226.7)	71.5 (11.1)	1.7 (0.6)

ms = milliseconds; *n* = number.

## Data Availability

The tDCS and ERP data are shown in this report. Additional data are available from the authors upon reasonable request.

## References

[1] Butler M.G., Lee P.D.K., Whitman B. (2006). Management of Prader-Willi Syndrome.

[2] Cassidy S.B., Schwartz S., Miller J.L., Driscoll D.J. (2011). Prader-Willi syndrome. Genet. Med..

[3] Butler M.G., Hartin S.N., Hossain W.A., Manzardo A.M., Kimonis V., Dykens E., Gold J.A., Kim S.-J., Weisensel N., Tamura R. (2019). Molecular genetic classification in Prader-Willi syndrome: A multisite cohort study. J. Med. Genet..

[4] Butler M.G., Miller J.L., Forster J.L. (2019). Prader-Willi Syndrome—Clinical Genetics, Diagnosis and Treatment Approaches: An Update. Curr. Pediatr. Rev..

[5] Butler M.G., Thompson T. (2000). Prader-Willi Syndrome. Endocrinologist.

[6] Butler M.G. (2006). Management of obesity in Prader-Willi syndrome. Nat. Clin. Pr. Endocrinol. Metab..

[7] Rolls E.T. (2000). The orbitofrontal cortex and reward. Cereb. Cortex.

[8] Rolls E.T. (2005). Taste, olfactory, and food texture processing in the brain, and the control of food intake. Physiol. Behav..

[9] Holsen L.M., Savage C.R., Martin L.E., Bruce A.S., Lepping R.J., Ko E., Brooks W.M., Butler M.G., Zarcone J.R., Goldstein J.M. (2011). Importance of reward and prefrontal circuitry in hunger and satiety: Prader–Willi syndrome vs simple obesity. Int. J. Obes..

[10] Zhang Y.E., Wang J., Zhang G., Zhu Q., Cai W., Tian J., Miller J.L., Wen X., Ding M., Gold M.S. (2015). The neurobiological drive for overeating implicated in Prader–Willi syndrome. Brain Res..

[11] Honea R.A., Holsen L.M., Lepping R.J., Perea R., Butler M.G., Brooks W.M., Savage C.R. (2012). The neuroanatomy of genetic subtype differences in Prader-Willi syndrome. Am. J. Med Genet. Part B Neuropsychiatr. Genet..

[12] Fregni F., Pascual-Leone A. (2007). Technology Insight: Noninvasive brain stimulation in neurology—Perspectives on the therapeutic potential of rTMS and tDCS. Nat. Clin. Pr. Neurol..

[13] Nitsche M.A., Paulus W. (2000). Excitability changes induced in the human motor cortex by weak transcranial direct current stimulation. J. Physiol..

[14] Vöröslakos M., Takeuchi Y., Brinyiczki K., Zombori T., Oliva A., Fernández-Ruiz A., Kozák G., Kincses Z.T., Iványi B., Buzsáki G. (2018). Direct effects of transcranial electric stimulation on brain circuits in rats and humans. Nat. Commun..

[15] Van Boekholdt L., Kerstens S., Khatoun A., Asamoah B., Mc Laughlin M. (2021). tDCS peripheral nerve stimulation: A neglected mode of action?. Mol. Psychiatry.

[16] Adair D., Truong D., Esmaeilpour Z., Gebodh N., Borges H., Ho L., Bremner J.D., Badran B.W., Napadow V., Clark V.P. (2020). Electrical stimulation of cranial nerves in cognition and disease. Brain Stimul..

[17] Fregni F., Ligouri P., Fecteau S., Nitsche M.A., Pascual-Leone A., Boggio P.S. (2008). Cortical Stimulation of the Prefrontal Cortex With Transcranial Direct Current Stimulation Reduces Cue-Provoked Smoking Craving. J. Clin. Psychiatry.

[18] Boggio P.S., Liguori P., Sultani N., Rezende L., Fecteau S., Fregni F. (2009). Cumulative priming effects of cortical stimulation on smoking cue-induced craving. Neurosci. Lett..

[19] Fregni F., Orsati F., Pedrosa W., Fecteau S., Tome F.A., Nitsche M.A., Mecca T., Macedo E.C., Pascual-Leone A., Boggio P.S. (2008). Transcranial direct current stimulation of the prefrontal cortex modulates the desire for specific foods. Appetite.

[20] Goldman R.L., Borckardt J.J., Frohman H.A., O’Neil P.M., Madan A., Campbell L.K., Budak A., George M.S. (2011). Prefrontal cortex transcranial direct current stimulation (tDCS) temporarily reduces food cravings and increases the self-reported ability to resist food in adults with frequent food craving. Appetite.

[21] Bravo G.L., Poje A.B., Perissinotti I., Marcondes B.F., Villamar M.F., Manzardo A.M., Luque L., Lepage J.-F., Stafford D., Fregni F. (2015). Transcranial direct current stimulation reduces food-craving and measures of hyperphagia behavior in participants with Prader-Willi syndrome. Am. J. Med. Genet. Part B Neuropsychiatr. Genet..

[22] Azevedo C.C., Trevizol A.P., Gomes J.S., Akiba H., Franco R.R., Simurro P.B., Ianni R.M., Grigolon R.B., Blumberger D.M., Dias A.M. (2020). Transcranial Direct Current Stimulation for Prader-Willi Syndrome. J. ECT.

[23] Szabo-Reed A.N., Breslin F.J., Lynch A.M., Patrician T.M., Martin L.E., Lepping R.J., Powell J.N., Yeh H.-W., Befort C.A., Sullivan D. (2015). Brain function predictors and outcome of weight loss and weight loss maintenance. Contemp. Clin. Trials.

[24] Szabo-Reed A.N., Martin L.E., Hu J., Yeh H.-W., Powell J., Lepping R.J., Patrician T.M., Breslin F.J., Donnelly J.E., Savage C.R. (2020). Modeling interactions between brain function, diet adherence behaviors, and weight loss success. Obes. Sci. Pr..

[25] Lang P.J., Bradley M.M., Cuthbert B.N. (1997). International Affective Picture System (IAPS): Affective Ratings of Pictures and Instruction Manual.

[26] Palm U., Reisinger E., Keeser D., Kuo M.-F., Pogarell O., Leicht G., Mulert C., Nitsche M.A., Padberg F. (2013). Evaluation of Sham Transcranial Direct Current Stimulation for Randomized, Placebo-Controlled Clinical Trials. Brain Stimul..

[27] Delorme A., Makeig S. (2004). EEGLAB: An open source toolbox for analysis of single-trial EEG dynamics including independent component analysis. J. Neurosci. Methods.

[28] Lopez-Calderon J., Luck S.J. (2014). ERPLAB: An open-source toolbox for the analysis of event-related potentials. Front. Hum. Neurosci..

[29] Chang C.-Y., Hsu S.-H., Pion-Tonachini L., Jung T.-P. (2020). Evaluation of Artifact Subspace Reconstruction for Automatic Artifact Components Removal in Multi-Channel EEG Recordings. IEEE Trans. Biomed. Eng..

[30] Delorme A., Palmer J., Onton J., Oostenveld R., Makeig S. (2012). Independent EEG Sources Are Dipolar. PLoS ONE.

[31] Chaumon M., Bishop D.V., Busch N.A. (2015). A practical guide to the selection of independent components of the electroencephalogram for artifact correction. J. Neurosci. Methods.

[32] Nolan H., Whelan R., Reilly R. (2010). FASTER: Fully Automated Statistical Thresholding for EEG artifact Rejection. J. Neurosci. Methods.

[33] Cohen J. (1988). Power Analysis for the Behavioral Sciences.

[34] Donkers F.C., Van Boxtel G.J. (2004). The N2 in go/no-go tasks reflects conflict monitoring not response inhibition. Brain Cogn..

[35] Key A.P.F., Dykens E.M. (2008). ‘Hungry Eyes’: Visual processing of food images in adults with Prader-Willi syndrome. J. Intellect. Disabil. Res..

[36] Stauder J.E.A., Boer H., Gertis R.H.A., Tummers A., Whittington J., Curfs L.M.G. (2005). Differences in behavioral phenotype between parental deletion and maternal uniparental disomy in Prader-Willi syndrome: An ERP study. Clin. Neurophysiol..

[37] Lapenta O.M., Di Sierve K., De Macedo E.C., Fregni F., Boggio P.S. (2014). Transcranial direct current stimulation modulates ERP-indexed inhibitory control and reduces food consumption. Appetite.

[38] Rafi S.K., Butler M.G. (2020). The 15q11.2 BP1-BP2 Microdeletion (Burnside–Butler) Syndrome: In Silico Analyses of the Four Coding Genes Reveal Functional Associations with Neurodevelopmental Disorders. Int. J. Mol. Sci..

[39] Van Der Meer D., Sønderby I.E., Kaufmann T., Walters G.B., Abdellaoui A., Ames D., Amunts K., Andersson M., Armstrong N.J., Writing Committee for the ENIGMA-CNV Working Group (2020). Association of Copy Number Variation of the 15q11.2 BP1-BP2 Region With Cortical and Subcortical Morphology and Cognition. JAMA Psychiatry.

[40] Yang X., Lin Y., Gao M., Jin X. (2018). Effect of Modulating Activity of DLPFC and Gender on Search Behavior: A tDCS Experiment. Front. Hum. Neurosci..

